# Growth Mixture Modeling of Depression Symptoms Following Traumatic Brain Injury

**DOI:** 10.3389/fpsyg.2017.01320

**Published:** 2017-08-22

**Authors:** Rapson Gomez, Clive Skilbeck, Matt Thomas, Mark Slatyer

**Affiliations:** ^1^School of Health Sciences, Federation University, Ballarat VIC, Australia; ^2^Psychology, School of Medicine, University of Tasmania, Hobart TAS, Australia; ^3^School of Psychology, Charles Sturt University, Bathurst NSW, Australia

**Keywords:** latent class growth modeling, traumatic brain injury, depression, outcome

## Abstract

Growth Mixture Modeling (GMM) was used to investigate the longitudinal trajectory of groups (classes) of depression symptoms, and how these groups were predicted by the covariates of age, sex, severity, and length of hospitalization following Traumatic Brain Injury (TBI) in a group of 1074 individuals (696 males, and 378 females) from the Royal Hobart Hospital, who sustained a TBI. The study began in late December 2003 and recruitment continued until early 2007. Ages ranged from 14 to 90 years, with a mean of 35.96 years (*SD* = 16.61). The study also examined the associations between the groups and causes of TBI. Symptoms of depression were assessed using the Hospital Anxiety and Depression Scale within 3 weeks of injury, and at 1, 3, 6, 12, and 24 months post-injury. The results revealed three groups: low, high, and delayed depression. In the low group depression scores remained below the clinical cut-off at all assessment points during the 24-months post-TBI, and in the high group, depression scores were above the clinical cut-off at all assessment points. The delayed group showed an increase in depression symptoms to 12 months after injury, followed by a return to initial assessment level during the following 12 months. Covariates were found to be differentially associated with the three groups. For example, relative to the low group, the high depression group was associated with more severe TBI, being female, and a shorter period of hospitalization. The delayed group also had a shorter period of hospitalization, were younger, and sustained less severe TBI. Our findings show considerable fluctuation of depression over time, and that a non-clinical level of depression at any one point in time does not necessarily mean that the person will continue to have non-clinical levels in the future. As we used GMM, we were able to show new findings and also bring clarity to contradictory past findings on depression and TBI. Consequently, we recommend the use of this approach in future studies in this area.

## Introduction

Traumatic brain injury (TBI) is among the leading causes of death and disability in adults. Many studies have shown high levels of depression symptoms and mood disorders among individuals with TBI (e.g., Hoofien et al., 2001; Fleminger et al., 2003; O’Connor et al., 2005; Whelan-Goodinson et al., 2009; Rapoport, 2010), although existing data also reflect inconsistencies. Fleminger et al. (2003) found that 20 to 40% of individuals with TBI showed signs of depression in the first year. Koponen et al. (2011) reported a rate of 15.8%, and Deb et al. (1999) reported a rate of 13.9%. Kreutzer et al. (2001) noted that 42% met criteria for major depression at 30 months post-injury. Also, in a longitudinal study involving individuals with traumas from occupational injuries, Wei et al. (2017) found rates of 9.2 and 2.0% at 6 years and 1 year, respectively, post-injury. Besides such wide inter-individual differences, there are also data showing wide intra-individual differences in levels of depression across time. As an example, Jorge et al. (2005) found that 40% of those who were depressed just after injury were no longer depressed 12 months later, while 18% without depression just after injury developed depression 12 months later.

Existing evidence indicates that generally the outcomes of TBI are related to the combined effects of pre-morbid, injury-related, and post-injury factors. Among the pre-morbid factors are age, sex, personality, social role, social support, education, IQ, psychiatric and substance abuse problems prior to injury, and psychological coping liabilities (Kay et al., 1992; MacMillan et al., 2002). Injury-related factors have included length of coma, duration of post-traumatic amnesia (PTA), and types of brain injury (Levin et al., 1982; Ruff et al., 1993). There are data showing higher rates with being older in age (Levin et al., 2005; Rao et al., 2010; Skilbeck et al., 2011), being female (Van Reekum et al., 1995; Demakis et al., 2010; Skilbeck et al., 2011), having a frontal subdural haematoma (Levin et al., 2005; Rao et al., 2010), higher initial severity of injury and PTA (Van Reekum et al., 1995), and higher disability post-injury (Demakis et al., 2010). In the longitudinal study involving individuals with traumas from occupational injuries, Wei et al. (2017) found that after adjustment for family and social factors, length of hospitalization post-injury, affected physical appearance, repeated occupational injuries, unemployment, and number of quit jobs after the injury contributing to higher depression rates.

In summary, although most studies have reported high rates of depression disorders post-TBI, the findings appear to be inconsistent in terms of their rates (Kreutzer et al., 2001; Whelan-Goodinson et al., 2009). Whelan-Goodinson et al. (2009) have noted that previous studies have reported rates from 10 to 46% for current depression, and 14 to 77% for various time points following TBI. Existing data also suggest a high degree of individual differences and fluctuation in depressive disorder rates across time and indicate that depression rates are likely to be moderated by pre-morbid and injury-related factors. These findings highlight the need for longitudinal studies that take account of inter and intra individual differences for depression levels, and also control for potential covariates that can influence depression levels.

A method that can fulfill these requirements is an extension of the conventional Latent Growth Analysis (LGA), called Growth Mixture Modeling (GMM, Muthén and Muthén, 2000; Muthén, 2004, 2006). The current study used GMM to identify groups of TBI survivors with different growth trajectories for depression, and the characteristics of these groups. In both LGA and GMM, the growth trajectories of an outcome measure that is repeatedly assessed at three or more interval points across time is modeled (Muthén, 2004). Each growth trajectory is characterized in terms of continuous scores for intercept (I), and linear slope parameter (S). With four or more data points the trajectory can also be characterized in terms of non-linear or quadratic parameter (Q). Unlike LGA, GMM does not assume that participants belong to a single homogeneous population. By relaxing this assumption, GMM is able to identify meaningful or naturally occurring homogenous subpopulations or classes (Muthén and Muthén, 2000; Muthén, 2004). To enable this, GMM includes a latent categorical variable, referred to as ‘class,’ which is regressed on the growth factors. This parameterization permits the entire sample to be separated into more homogeneous subgroups or classes, based on close similarities in growth trajectories. When there are no covariates in the model it is referred to as an unconditional model.

Growth Mixture Model contains both latent growth variables η and a latent categorical class variable c for *c* = 1, 2,...,K. Using these notations, the equation [taken extensively from Reinecke (2006)] for the unconditional GMM can be expressed as:

$$\begin{array}{l} \gamma \text{tk}=\lambda 1\text{tk}\eta 1\text{k}+\lambda 2\text{tk}\eta 2\text{k}+\varepsilon \text{tk}\\ \eta \text{1k = }\alpha \text{1k + }\zeta \text{1k}\\ \eta \text{2k = }\alpha \text{2k + }\zeta \text{2k, where} \end{array}$$

γtk = the manifest variable measured in wave t and class k,
λ1tk = initial level factor (intercept); (constrained to 1 for all γtk)
λ2tk = linear growth factor (restricted to represent linear growth: λ2tk = 0, 1, 2,...T),
εtk = random error term.

As will be noticed, the latent variables in this model are described by their class specific mean (α1k, α2k) and variance (ζ1k, ζ2k) scores.

Growth Mixture Modeling also allows the inclusion of covariates. This is referred to as a conditional model. By regressing the class variable on the covariates, GMM allows the examination of how the classes are influenced and predicted by the covariates. In the case of a conditional model, the above equation includes the exogenous latent variables ξn, and is ηmk = Ak + Γkξnk + ζmk, where matrix Ak contains the levels and slopes within k-classes, and matrix Γk refers to the regressions of ξn within the kth class (Reinecke, 2006). If desired, it is also possible to extend the GMM analyses to include distal outcome variables. This would indicate how the classes differ for the modeled distal outcome variables. It is also possible to determine how the covariates and the within-class growth factors predict the distal outcome scores for each class (Reinecke, 2006).

To date at least two published studies have been reported that have used GMM to examine the trajectory classes for symptoms related to depression following other forms of traumatic injuries (deRoon-Cassini et al., 2010; Bonanno et al., 2012). Additionally, at least one study, using latent class growth curve analysis (LCGA; Nagin, 1999) has examined depression following TBI (Bombardier et al., 2016). Unlike GMM, LCGA constraints the within class variances for the growth parameters to zero. The participants in the deRoon-Cassini et al. (2010) study were primarily trauma survivors injured by automobile crash, assault, industrial and home accidents, and falls, without brain or spinal cord injuries or neurological and cognitive impairment. The levels of depression symptoms were measured at four times across the first 6 months after surgery (during hospitalization, and at 1, 3, and 6 months post-surgery). Findings indicated four latent classes. They were chronic (high level over the entire 6 months), delayed (moderate initial level that increased to high level by the sixth month), recovered (high initial level that decreased to low level by the sixth month), and resilient (low level over the entire 6 months). The latter formed the largest class (60%). The study by Bonanno et al. (2012) examined trajectories of depression following spinal cord injury. Participants were assessed within 6 weeks of injury, and at 3 months, 1 year, and 2 years from the time of injury. Results revealed the four classes reported by deRoon-Cassini: chronic, delayed, recovered, and resilient. The largest class was resilient (66.1%). Bombardier et al. (2016) examined depression trajectories over a 1 year interval (four time points) following mild to severe TBI. Both the unconditional and conditional models resulted in four trajectory groups: low depression, delayed depression, depression recovery, and persistent depression, with low depression being the largest class. Compared to the low depression group, the other three groups were more likely to have pre-injury history of mental health disorders, drug and alcohol abuse, and on Medicare rather than commercial insurance. Also of relevance to this study, Juengst et al. (2015) used LCGA to examine life satisfaction trajectories over a 5 year interval (three time points) following moderate to severe TBI. Both the unconditional and conditional models resulted in four trajectory groups: stable satisfaction, stable dissatisfaction, initial dissatisfaction improving, and initial satisfaction declining, with stable satisfaction being the largest group. Age, depressive symptoms, cognitive disability, and life role participation as a worker, leisure participant, and/ or religious participant at 1 year post-injury significantly predicted trajectory group membership.

To date, no studies have used GMM to examine the longitudinal trajectories of depression following TBI. Such studies would be valuable, as the findings would have important implications for responding to the mental health needs of TBI survivors. The first aim of the current study was to use GMM to examine the longitudinal trajectories (over a 2-year period) of depression immediately following TBI. As there are data showing higher depression rates vary with age, being female, and more severe injury (reviewed above), the second aim of the study was to examine these longitudinal trajectories, controlling for the effects of age, sex, PTA, and additionally, number of days in hospital following injury, and how these covariates were differentially associated with the groups/classes. A third aim was to examine how the classes differed in terms of cause of TBI. Although it would have been possible to also have cause of TBI as a covariate to predict the growth trajectories, this was not possible because the increased complexity of doing this in the conditional model led to model inconvergence. Based on the findings reported by deRoon-Cassini et al. (2010), Bonanno et al. (2012), and Bombardier et al. (2016), we expected to find the following same four trajectory classes: low (low levels of depression over 24 months), high (high levels of depression over 24 months), delayed (moderate initial level of depression with increasing levels across the 24 months), and recovered (high initial level of depression with decreasing levels across the 24 months). Additionally, given past findings, we predicted that PTA and shorter period of hospitalization would be associated positively with the high and delayed depression classes.

## Materials and Methods

### Participants

**Table 1** provides background information of participants, the sample comprising 1074 individuals (696 males, and 378 females), with age ranging from 14 to 90 years, who sustained a TBI and were recruited from the Royal Hobart Hospital, as part of a population study of TBI in Southern Tasmania. All individuals on the TBI register, regardless of age were included in the study. The mean age of these individuals was 35.96 years (*SD* = 16.61). The wide age range was not seen as problematic as we controlled for age in the GMM. The mean estimated premorbid IQ was 99.23 (10.581; *N* = 718), and 50.9% were currently in a relationship, and 49.1% were not (*N* = 919). TBI cause was established for 1064 participants: motor vehicle accident (39.2%), assault (27.3%), fall (20.0%), sport (7.0%), and others (6.4%). The mean number of hours in hospital immediately following injury was 5.70 (*SD* = 13.792; *N* = 1005), and the mean PTA score was 2.26 h (*SD* = 6.672; *N* = 1037). The percentages of very mild, mild, moderate, and severe TBI were 51.8, 19.9, 26.6, and 1.7, respectively, based on duration of PTA (<5 min = very mild, between 5 and 60 min = mild, 1–24 h = moderate, 1+ days = severe; Jennett and Teasdale, 1981). **Table 2** provides the distribution of causes of TBI by age [adolescent/emerging adults (14 to ≤25), adult (25 to ≤65), and older adult (≥65 to 90)], sex, and TBI severity (PTA ≤ 3 days, and PTA > 3 days). The latter categorization of PTA was based on the criteria provided for the measures used for assessing PTA in the current study (see “Post-traumatic amnesia” subsection below), i.e., to be considered out of PTA, an individual must have perfect performances across 3 consecutive days, with the end of PTA being the first of the 3 days.

**Table 1:** Background data of participants in the study.

Variable	Statistics	Score
Age (*N* = 1074)	Mean (*SD*)	35.96 (16.608)
Sex (*N* = 1074)		
Male	Frequency (%)	696 (64.8)
Female	Frequency (%)	378 (35.2)
IQ (*N* = 718)	Mean (*SD*)	99.23 (10.581)
Current relationship (*N* = 919)		
In	Frequency (%)	468 (50.9)
Not in	Frequency (%)	451 (49.1)
Causes of TBI (*N* = 1064)		
Motor vehicle accident (all)	Frequency (%)	417 (39.2)
Fall	Frequency (%)	213 (20.0)
Assault	Frequency (%)	291 (27.3)
Sport	Frequency (%)	75 (7.0)
Other	Frequency (%)	68 (6.4)
Severity of TBI (based on duration of PTA (*N* = 1037)		
Very mild	Frequency (%)	537 (51.8)
Mild	Frequency (%)	206 (19.9)
Moderate	Frequency (%)	276 (26.6)
At least severe	Frequency (%)	17 (1.7)
PTA (hours) (*N* = 1037)	Mean (*SD*)	2.256 (6.672)
# of Hosp days just after injury (*N* = 1005)	Mean (*SD*)	5.70 (13.792)

**Table 2:** Percentage distribution of causes by age, sex, and TBI severity.

		Cause				
Sex	Age category	1	2	3	4	5
**PTA ≤ 3 days**						
Female	Adolescent/emerging adults	44.4	17.4	58.3	40	31.2
	Adult	46.6	55.4	41.7	60	68.8
	Older adult	9	27.2	0	0	0
Male	Adolescent/emerging adults	38.1	16.3	45.3	68	35.3
	Adult	55.8	67.3	53.7	32	58.8
	Older adult	6.1	16.3	1.1	0	5.9
**PTA > 3 days**						
Female	Adolescent/emerging adults	28.6	0	50	0	100
	Adult	67.9	66.7	50	100	0
	Older adult	3.6	33.3	0	0	0
Male	Adolescent/emerging adults	36.5	20	36	25	12.5
	Adult	61.9	66.7	64	75	75
	Older adult	1.6	13.3	0	0	12.5

*Age range for adolescent/emerging adults = 14 to ≤25, Adult = 25 to ≤65, Older Adult = ≥65 to 90. Cause: 1 = motor vehicle accident, 2 = fall, 3 = assault, 4 = sport, 5 = other*.

### Instruments

#### Depression

Depression was measured using the Hospital Anxiety and Depression Scale (HADS; Zigmond and Snaith, 1983), a self-report questionnaire developed to measure anxiety^1^ and depression among patients in non-psychiatric hospital settings. The depression scale has seven items assessing depression during the past week, with all items focussed on affective and cognitive symptoms rather than somatic symptoms. Each item is rated on a scale from 0 to 3, with higher scores indicating greater severity. The study used the total scores for the depression scale, possible scores ranging from 0 to 21. A recent study found that scores of 8 or more were clinically relevant (Olssøn et al., 2005). The reliability and validity of the HADS, including its ability to assess depression in primary and general populations have been established (Bjelland et al., 2002), and it has also been used in studies of acquired brain injury (Dawkins et al., 2006; Skilbeck et al., 2011). In the present study, the internal consistency (Cronbach’s alpha) for the depression scale within 3 weeks of injury was 0.83.

#### Premorbid IQ

The National Adult Reading Test (NART; Nelson, 1982) was used to obtain estimated premorbid IQ. The test requires subjects to read out 50 words that are irregular in terms of their grapheme–phoneme correspondences. The total number of words that are correctly pronounced is used to derive an estimated premorbid IQ. Nelson and O’Connell (1978) showed that the NART was a robust predictor of premorbid levels in 40 patients with frontal bilateral cortical atrophy.

#### Post-traumatic Amnesia (PTA)

For those admitted to hospital, the end of PTA was assessed using the Westmead PTA Scale (WPTAS; Shores et al., 1986). For those participants not admitted to hospital, PTA duration was subjectively estimated using the Galveston Orientation and Amnesia Test (GOAT; Levin et al., 1979). The WPTAS contains 12 simple items measuring orientation and recall or recognition of new information. The scale is administered daily. In order to be considered out of PTA, patients must obtain perfect (12/12) performances across 3 consecutive days, with the end of PTA being the first of the 3 days. The scale has been validated in several studies, including PTA in adults and children with TBI (Shores et al., 1986; Marosszeky et al., 1993; Ponsford et al., 2004). The GOAT is a 10-item scale that assesses orientation and memory for events preceding and following TBI and is well established (Levin et al., 1979; Sherer et al., 2002). Our classification of severity of TBI was based upon length of PTA, as described by the Veterans Administration/Department of Disability Clinical Practice Guideline taxonomy (Group, 2009) as is common practice.

### Procedure

Ethical approval for this study was obtained from the Tasmanian Human Research Ethics Committee (H7116). Potential participants were approached as soon as possible after their attendance at the Emergency Department of the Royal Hobart Hospital (RHH). Written informed consent was obtained from those able to provide it at that time. For those patients admitted in coma or still in PTA, initial consent was obtained from next of kin; these participants were then approached to provide written informed consent as soon as they had regained consciousness and their PTA had ended. They were provided with an information leaflet about the study, with assurance that their decision was voluntary, and that their decision would in no way affect their treatment. For individuals under 18 years of age, consent was also obtained from their parents. All consenting participants were provided with the HADS questionnaire to complete. During this and for all other subsequent data points the questionnaires were administered during assessments at the RHH. Among other measures, depression was measured six times: within 3 weeks of injury, and at 1, 3, 6, 12, and 24 months post-injury. If a patient had the first assessment after 3 weeks, then the scores were considered 1 month data. One-month was recorded if the scores were obtained between 21 and 38 days post-TBI, 3-month if scores were obtained between 61 and 121 days post-TBI, 6-months if scores were obtained between 122 and 240 days post-TBI, 12-months if scores were obtained between 300 and 420 days post-TBI, and 24-months if scores were obtained between 631 and 900 days post-TBI. Response rates for 0, 1, 3, 6, 12, and 24 months for HADs depression were 97.67, 55.59, 63.04, 59.22, 54.56, and 45.34%, respectively, and at least 69.9% of participants had three or more data points, and 32% all six data points.

### Statistical Analysis

Statistical analyses were conducted using M*plus* Version 6.12 (Muthén and Muthén, 2010), and the analyses used robust maximum likelihood estimation. Full information maximum likelihood was applied to deal with missing values. This procedure, which assumes that data are unrelated to the outcome variables or missing at random, is a widely accepted approach for handling missing data (Schafer and Graham, 2002).

**FIGURE 1:**
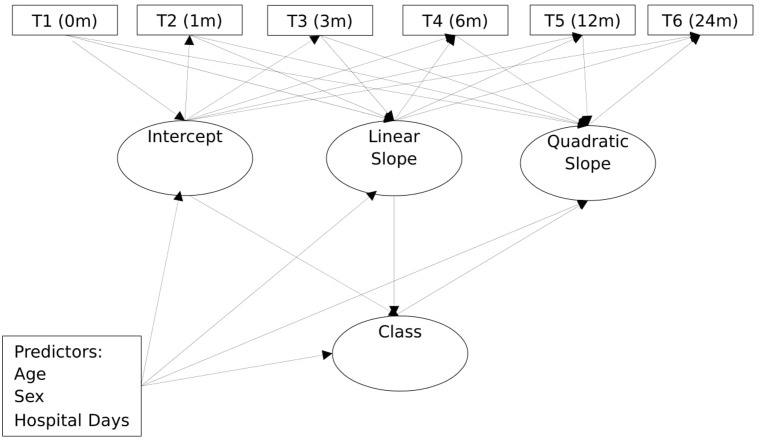
General diagram of the growth mixture model used in the study.

**Figure 1** shows a schematic path diagram of the model used for the analyses in the study. As shown, the model included a univariate latent growth curve with intercept (I), linear parameter (S) and the non-linear quadratic parameter (Q). The observed variables were the repeated outcome measure of depression, obtained at the six different time points: initial (T1), 1 month after injury (T2), 3 months after injury (T3), 6 months after injury (T4), 12 months after injury (T5), and 24 months after injury (T6). To capture classes in the sample, a categorical variable for group/class (C) was regressed on these growth parameters. Together, these components constituted the unconditional growth model. **Figure 1** also includes the covariates, thereby extending the unconditional growth model to a conditional growth model. The covariates were age, sex, PTA, and number of days in hospital immediately following injury. As shown, the latent class variable and the within class growth factors were regressed on the covariates.

The GMM analyses involved five steps. The first step involved running three variants of unconditional simple Latent Growth Model (where the entire sample is considered as belonging to a single homogenous group): with intercept only (no growth), intercept and slope (linear growth), and intercept, linear parameter, and quadratic parameter (non-linear growth). These models were compared using likelihood ratio chi-square statistics. The non-linear models provided the best fit. In the second step, we extended the non-linear models to compare 2- to 5-class unconditional (no covariates) GMMs to allow us to ascertain the optimum GMM model. For all these models, all growth factors were freely estimated. Initially, the variances of all growth factors were freely estimated within classes (non-invariant models). The model failed to converge. A series of alternate models constraining the variances of the growth parameters either alone or in pairs equal across the classes also failed to converge or provided inadmissible solutions. An admissible solution was obtained only when the variances for the intercepts and both slopes parameters for the classes were constrained equal across all classes. Given this, the GMM model was conducted with the growth parameter constrained invariant across the classes. All other M*plus* default specifications were retained.

It has been argued that once the optimum unconditional model has been established, it would be necessary to examine the parameters (such as the growth parameters and trajectories) in this model after controlling for potential covariates (Muthén, 2003). To enable this, the third step involved regressing class membership and the class-specific growth factors in the GMM model (with the growth parameter constrained invariant across the classes) on all the covariates simultaneously. This analysis also allowed for examination of how the classes were differentially associated with the covariates. As all these covariates were entered simultaneously, the differential association of each covariate was evaluated, controlling for the influences of the other covariates. As the latent class variable is categorical, the relationships of this variable with the covariates are multinomial logistical regression. Thus, the coefficients represent the log odds of being in the non-reference classes versus being in the reference class.

To date a number of approaches have been proposed to help determine the optimal number of latent classes in GMM. These procedures, which account for model complexity, included a number of information criteria, including the Akaike Information Criterion (AIC; Akaike, 1973), the Bayesian Information Criterion (BIC; Schwarz, 1978), and the sample-size adjusted BIC (ABIC; Sclove, 1987). Other useful indices are the Lo–Mendell–Rubin (LMR) statistic (Lo et al., 2001) and the adjusted Lo-Mendell-Rubin likelihood-ratio test (ALRT). Both LMR and ALRT test a model with *K* classes versus a model with *K-1* classes. A significant *p*-value indicates that the model with *K* classes is better than the model with *K-1* classes. Usually, a non-significant *p*-value indicates that the model with *K* classes is not an improvement over the model with *K-1* classes. In the current study, the optimal numbers of trajectory classes were determined using all the information fit statistics and the LMR and ALRT, and also the parsimony, theoretical justification, and interpretability of the models (Bauer and Curran, 2003; Muthén, 2003; Rindskopf, 2003). As used in past research (e.g., Ranby et al., 2012), when there was a discrepancy between the LMR test and the information fit statistics, the BIC was given greater consideration as it has been shown to outperform the other information criteria (Nylund et al., 2007). In addition, as suggested by Jung and Wickrama (2008), other considerations were successful convergence, high entropy value (near 1.0), at least 1% of total count in a class, and high posterior probabilities (near 1.0).

## Results

### Missing Values

As noted, full information maximum likelihood was applied to deal with missing values. The lowest covariance coverage for each pair of variables (obtained using M*plus*) was 0.291. As this value is above the minimum threshold of 0.10 for model convergence, the missing values are within acceptable limits for the analyses.

### Unconditional GMM Model

**Table 3** provides the AIC, BIC, ABIS, LMR, ALRT and entropy for the 1-, 2-, 3-, and 4-class solutions. As will be noticed, the AIC, BIC and ABIS decreased from the 1- to 2- to 3- to 4-class models. For all indices, the decrements were relatively larger from the 1- to 2-class models, and 2 to 3-class models, compared to the 3- to 4-class models. The LMR and ALRT values for the 1-, 2-, and 3-class models were significant, whereas these values were not significant for the 4-class model. Also, the 4-class model was relatively uninformative, as it simply separated one of the classes (the delayed depression class, described below) into two, based on intercept values (i.e., difference in intercept, but little difference in the trajectory). Given that past studies with other forms of traumatic injuries have indicated four trajectory groups/classes for depression (deRoon-Cassini et al., 2010; Bonanno et al., 2012), it was decided to examine a 5-class model. As shown in **Table 3**, the decrement of the AIC, BIC and ABIS values from the 3- to 5-class models were relatively large compared to the 4- to 5-class models. Also, the LMR and ALRT values for the 5-class model were not significant. The overall classification accuracy (entropy) for the 3-class models was high (0.756), and slightly higher than the 4-class (0.745) and 5-class (0.701) models. The entropy value for their 3-class model was higher than that for the 1- and 2-class models. The posterior probabilities of individuals correctly classified in the three classes were also high (0.922, 0.785, and 0.791). Thus, based on statistical grounds, the 3-class models can be considered optimum. Another reason for accepting this model is that the three trajectories found were comparable to three trajectories observed in GMM studies involving other forms of trauma (deRoon-Cassini et al., 2010; Bonanno et al., 2012), as described below.

**Table 3:** Fit Indices for unconditional 1- to 4-class growth mixture models (*N* = 1074).

Fit Indices	1-class	2-classes	3-classes	4-classes	5-classes
AIC	21077.797	20849.010	20733.827	20634.866	20625.665
BIC	21137.547	20928.677	20833.410	20754.365	20735.081
Adjusted BIC	21099.433	20877.858	20769.886	20678.137	20666.147
Entropy	–	0.746	0.756	0.745	0.701
LMR *p*-value	–	0.0210	0.0303	0.2868	0.3805
ALRT *p*-value	–	0.0239	0.0336	0.2959	0.3881

*AIC, Akaike information criterion; BIC, Bayesian information criterion; Adjusted BIC, sample size-adjusted Bayesian information criterion; LMR, Vuong-Lo–Mendell–Rubin test; ALRT, Adjusted Lo–Mendell–Rubin test*.

### Trajectories and Mean Scores of Growth Factors, 3-Class Unconditional Model

**FIGURE 2:**
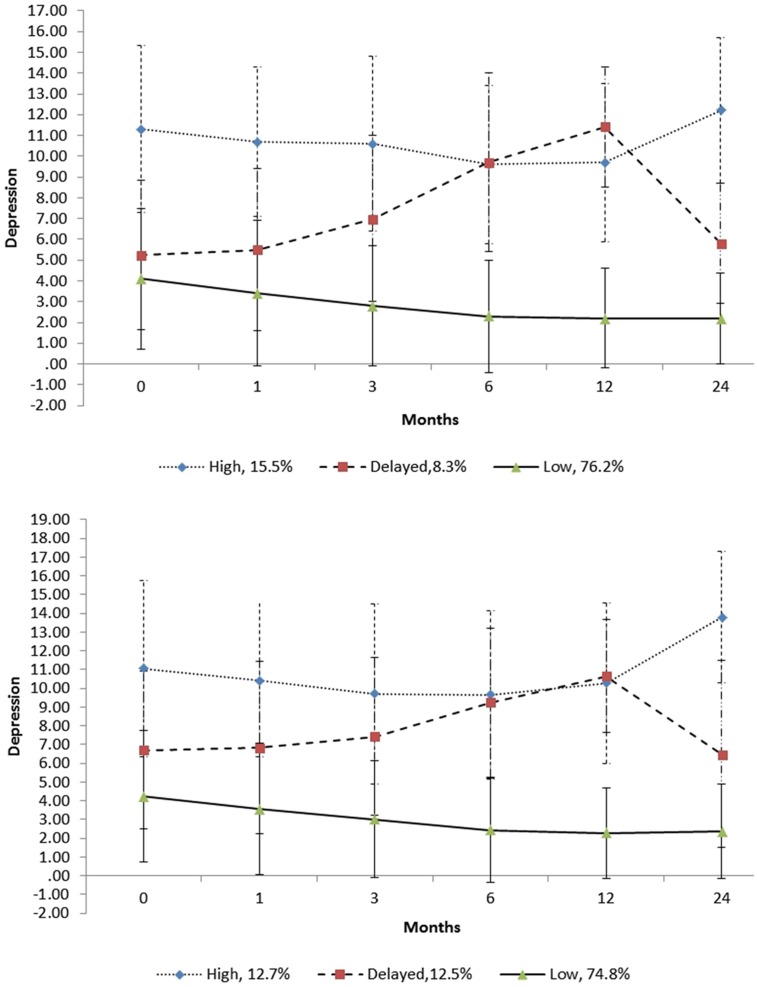
Trajectories of the unconditional (above) and conditional (below) 3-class models. The error bars are ±1 standard deviation intervals.

**Table 4** shows the parameter estimates of the growth factors for the classes in the unconditional 3-class model. **Figure 2** shows the trajectories for the classes in the unconditional 3-class model. As will be noticed in this figure, one of the classes (comprising 76.2% of the sample) had relatively low initial depression scores (around 4), with the trajectory remaining relatively stable and well below the clinically relevant cut-off score (≥8) over virtually the entire 24 months. This class can be considered a low depression class. A second class (comprising 8.3% of the sample) had moderate initial depression. As shown in **Figure 2**, there was a notable score increment in the slope in the first 12 months, rising from around 6 at the initial level to 11 at 12 months, followed by a sharp decrement, (the level at 24 months being close to the initial level of 6). Although the trajectory for this class was below the clinically relevant cut-off score level initially and at 24 months, it was above the clinically relevant cut-off score over most of the 24 months and can be considered a delayed depression class. The third class (comprising 15.5% of the sample) had the highest initial depression score (around 11), with some decrease in depression over the first 6 months followed by a moderately sharp increase over the next 18 months. At 6 months, the means score was approximately 9, and at 24 months, it was around 12. For this class, the trajectory remained well above the clinically relevant cut-off score over the entire 24 months. This group/class can be considered a high depression class.

**Table 4:** Mean scores for the growth factors of the 3-class unconditional and conditional model.

Growth Factors	Class 1 (High)		Class 2 (Delayed)		Class 3 (Low)	
	Mean	*SE*	Mean	*SE*	Mean	*SE*
**Unconditional Model (*N* = 1074)**						
Intercept	10.575^∗∗∗^	0.611	5.270^∗∗∗^	0.742	3.840^∗∗∗^	0.253
Linear parameter	-0.179^∗∗∗^	0.093	0.971^∗∗∗^	0.175	-0.247^∗∗∗^	0.022
Quadratic parameter	0.011^∗∗∗^	0.004	-0.039^∗∗∗^	0.007	0.008^∗∗∗^	0.001
**Conditional Model (*N* = 975)**						
Intercept	9.529^∗∗∗^	0.611	6.614^∗∗∗^	0.742	3.933^∗∗∗^	0.253
Linear parameter	-0.141^∗∗∗^	0.093	0.677^∗∗∗^	0.175	-0.255^∗∗∗^	0.022
Quadratic parameter	0.013^∗∗∗^	0.004	-0.028^∗∗∗^	0.007	0.009^∗∗∗^	0.001

*Since the variances of respective intercepts, linear parameters, and quadratic parameters were set invariant across the classes their values were the same across classes. The variance (*SE*) for intercepts, linear parameters, and quadratic parameters for the conditional model were 7.671 (1.256), 0.015 (0.003), and 0.001 (0.001), respectively. ^∗∗∗^*p* < 0.001*.

Although not shown in **Figure 2**, all three trajectories did not overlap at any point over the 24 months when their standard error (*SE*) scores were considered. As shown in **Figure 2**, when their standard deviation (SD) scores were examined, there was notable overlap of the trajectories, implying there was considerable fluctuation of the depression scores of individuals within each class. As shown in **Figure 2**, for the delayed depression and high depression classes, their ±1 *SD* scores were spread below and above the clinical cut-off score of 8 over the 24-month period. Also, as shown, the +1 *SD* scores over this period for the low depression class were below this clinical cut-off score.

### Trajectories and Mean Scores of Growth Factors for the 3-Class Conditional Model

Because of missing data on the covariates, the sample size for the condition model was reduced to 975. The entropy value (measure of classification accuracy for this model was 0.752), and the posterior probabilities for the four classes were also high (0.918, 0.778, and 0.837). The entropy can be considered high since it was >0.70 (Juengst et al., 2015).

**Table 4** includes the parameter estimates of the growth factors for the classes in the conditional 3-class model. **Figure 2** includes the trajectories for the classes in the conditional 3-class model. Comparisons of these trajectories with the 3-class unconditional model suggest a high degree of similarity. However, a difference worth noting is that the delayed class in the unconditional model had steeper linear trajectory than that in the unconditional model. There was also some difference in the percentages of individuals in the classes, with these being 74.7, 12.5, and 12.7% for the low, delayed and high classes, respectively, for the conditional model. **Table 4** includes the parameter estimates of the growth factors for the classes in the conditional 3-class model.

### Prediction of Class Membership

**Table 5** shows the logistic coefficients for the regression of latent factor for class on the predictors. With the low class as the reference class, the coefficient for number of days in hospital was significant and negative for the delayed class. For the high class, the coefficient for PTA was significant and positive and the coefficient for number of days in hospital was significant and negative. These findings suggest that compared to the low class, the delayed and high classes spent fewer days in hospital, and the high class had more severe TBI.

**Table 5:** Multinomial logistic regression for predictors of depression class membership (*N* = 975).

	Delayed			High		
Variables	Estimate (*SE*)	95 % CI	Mean (*SD*)	Estimate (*SE*)	95 % CI	Mean (*SD*)
Age	0.02 (0.01)	±0.021	41.46 (17.91)	-0.00 (0.01)	±0.016	35.88 (11.83)
Sex	-0.47 (0.46)	±0.904	1.55 (0.50)	0.21 (0.35)	±0.678	1.70 (0.46)
PTA	-0.03 (0.03)	±0.063	2.58 (7.23)	0.05^∗^ (0.02)	±0.045	3.33 (5.23)
Hospital days	-0.04^∗^ (0.02)	±0.029	3.42 (22.39)	-0.06^∗^ (0.03)	±0.057	2.36 (5.28)

*Low class served as the referent. OR, odds ratio; CI, confidence interval; PTA, post-traumatic amnesia; Hospital days = number of days from injury to first assessment. For sex, female = 1 and male = 2. The mean (*SD*) for the low class for age, sex, PTA, and hospital days were 35.53 (16.80), 1.66 (0.47), 2.05 (6.67), and 4.90 (12.30), respectively. ^∗^*p* < 0.05*.

Given the association between TBI and class membership, the association between severity of brain injury was examined, based on the duration of PTA via a 3-class conditional GMM without PTA as a covariate. The trajectories and class memberships of this model were close to the original 3-class conditional GMM. The class membership from 3-class conditional GMM without PTA as a covariate was used to examine how class member was associated with TBI severity. A 3 (class) × 4 (severity) χ^2^ analysis was not significant, χ^2^ (6, *N* = 975) = 41.61, *ns*, thereby indicating no association between class and TBI severity.

### Prediction of Growth Factors

**Table 6** shows the coefficients for the regression of the class specific growth factors on the predictors. For the high class, the intercept parameter was associated positively with age and PTA, and negatively with sex. The linear parameter was associated positively with age, sex, and hospital stay. The quadratic parameter was associated negatively with sex. For the delayed class, the intercept was associated negatively with age and PTA. The linear parameter was associated positively with PTA and negatively with number of days in hospital. The quadratic parameter has no significant association. For the low class, the intercept was associated negatively with sex, and positively with age PTA. The linear parameter was associated positively with sex, and the quadratic parameter was associated negatively with sex.

**Table 6:** Regression of growth factors on the covariates in the conditional 3-class model (*N* = 975).

	Intercept		Linear parameter		Quadratic parameter	
Predictors	Estimate	*SE*	Estimate	*SE*	Estimate	*SE*
**High**						
Age	0.158^∗∗^	0.058	0.016^∗^	0.007	-0.001	0.000
Sex	-3.954^∗∗^	1.290	0.633^∗∗^	0.229	-0.020^∗^	0.009
PTA	0.337^∗∗^	0.103	-0.012	0.009	0.000	0.000
Hospital days	-0.190	0.115	0.023^∗∗^	0.008	-0.001	0.000
**Delayed**						
Age	-0.096^∗∗∗^	0.024	0.007	0.006	0.000	0.000
Sex	0.678	1.423	-0.179	0.299	0.007	0.011
PTA	-0.164^∗^	0.080	0.042^∗∗∗^	0.011	-0.000	0.000
Hospital days	0.034	0.023	-0.010^∗^	0.004	0.000	0.000
**Low**						
Age	0.020^∗∗^	0.008	0.000	0.001	0.000	0.000
Sex	-1.271^∗∗∗^	0.351	0.122^∗^	0.050	-0.004^∗^	0.002
PTA	0.074^∗^	0.034	-0.003	0.005	0.000	0.000
Hospital days	-0.007	0.014	-0.001	0.002	0.000	0.000

*PTA, post-traumatic amnesia; Hospital days = number of days from injury to first assessment. For sex, female = 1 and male = 2. ^∗∗∗^*p* < 0.001, ^∗∗^*p* < 0.01, ^∗^*p* < 0.05*.

### Differential Associations of Classes with Cause of TBI

**Table 7** shows the frequencies in the low, delayed and high classes whose TBI was caused by motor vehicle accident, fall, assault, sport, and other. In *post hoc* analysis, a 3 (class) × 5 (cause) χ^2^ analysis was used to ascertain if the classes were differentially associated with cause of TBI. The χ^2^ was significant, χ^2^ (8, *N* = 967) = 41.61, *p* < 0.001, thereby indicating an association between classes and cause. Haberman’s (1978) standardized adjusted residuals statistic (HAR) was used to evaluate more closely this association. The low class had more individuals from fall (*N* = 157; HAR = 2.1, *p* < 0.05) and sport (*N* = 66; HAR = 3.8, *p* < 0.001) than expected by chance. (*N* for fall = 161, and *N* for sport = 54). The high class had more individuals from assault (*N* = 43; HAR = 3.0, *p* < 0.01) than expected by chance (*N* = 30), and the delayed class had more individuals from motor vehicle accident (*N* = 56; HAR = 4.3, *p* < 0.001) than expected by chance (*N* = 37).

**Table 7:** Percentages (%) and Haberman’s Standardized Adjusted Residuals (HAR) of individual with different causes of TBI in the classes for depression.

		MVA	Fall	Assault	Sport	Other
Low (*N* = 763)	%	276	157 (161)	172	203 (54)	66
	HAR		2.1^∗^		3.8^∗∗∗^	
Delayed (*N* = 94)	%	56 (37)	18	15	0	5
	HAR	4.3^∗∗∗^				
High (*N* = 110)	% HAR	47	14	43^∗^ (30) 3.0^∗∗^	2	4

*3 (class) × 5 (causes) χ^2^ (8, *N* = 967) = 41.61, *p* < 0.001 (suggests association between classes and causes). Values in parenthesis are expected frequencies. MVA, Motor vehicle accident. ^∗^*p* < 0.05, ^∗∗^*p* < 0.01, ^∗∗∗^*p* < 0.001*.

## Discussion

The current study used GMM to examine the longitudinal trajectory classes for depression over the 24-month period post-TBI. The sample comprising 1074 individuals (696 males, and 378 females), with age ranging from 14 to 90 years (mean age = 35.96 years, *SD* = 16.61 years) who sustained a TBI. The ratio of males to females in our sample (1:1.8), and the demographics response rates in terms of age, sex, TBI cause and severity are comparable to that reported in other epidemiological studies, conducted in Australian and internationally, (e.g., Hillier et al., 1997; Fortune and Wen, 1999). Also, the wide age range in our sample was not seen as problematic as we controlled for age in the GMM.

The unconditional model (without covariates) indicated that the optimum fitting model was the 3-class model. The trajectories for this model were characterized as low, high, and delayed. For the low class (comprising 76.2% of the sample), the trajectory remained well below the clinically relevant cut-off score of 8 for the entire 24 months. The trajectory for the high class (comprising 15.5% of the sample) remained well above the clinically relevant cut-off score for the entire 24 months. Despite this, it decreased slightly over the first 12 months, followed by a sharper increase for the remaining months. For the delayed class (comprising 8.3% of the sample), the trajectory increased continuously from just after injury to 12 months, and then decreased to approximately its initial level at 24 months. Except for the levels at around 12 months, the levels before and after were lower than the high class. However, the trajectory was around or above the clinically relevant cut-off score for the most of the 24 months.

The findings in this study showed that when *SD* scores were considered, there was notable overlap of the trajectories for both the conditional and unconditional models, thereby suggesting considerable fluctuation of the depression scores of individuals within each class. For the delayed depression and high depression classes, their ±1 *SD* scores were spread below and above the clinical cut-off score of 8 over the 24-month period, whereas the +1 *SD* scores for the low depression class were below this clinical cut-off score. These findings suggest that many individuals who experience elevated levels of depression following TBI are likely to demonstrate considerable fluctuation of depression over time, with levels falling below the clinical level.

The findings in the current are generally consistent with existing data in showing that most TBI survivors do not experience high depression, and in finding wide intra-individual differences in levels depression across time. For example, Jorge et al. (2005) found that 40% of those who were depressed just after injury were no longer depressed 12 months later, while 18% without depression just after injury developed depression 12 months later. The current findings also extend existing data, by applying a GMM procedure able to identify more meaningful or naturally occurring classes of individuals with depression. Also, as the GMM also included covariates, we were able to examine how these classes were influenced and predicted by the covariates.

The study examined how age, sex, PTA, and number of days in hospital immediately following injury influenced the growth trajectories of the classes. The findings showed only slight differences between the 3-class unconditional model and the 3-class conditional model (with covariates). Notably, for the delayed class, the rate of increase in the linear parameter was higher in the unconditional model than the conditional model. There were also differences in the number of individuals in the different classes. For the low, delayed and high conditional classes, the percentages were 74.7, 12.5, and 12.7%, respectively, compared to 76.2, 8.3, and 15.5%, respectively, for the unconditional classes. Thus, there were relatively fewer individuals in the high class and relatively more individuals in the delayed class in the conditional model compared to these classes in the unconditional model. These findings indicate that while the covariates impacted on the trajectory of the delayed class and on class membership, there was generally close correspondence between the trajectory classes for the unconditional and conditional models.

As part of this study, we examined how the covariates (age, sex, PTA, and number of days in hospital after injury) predicted the classes. Despite the comparability of the unconditional and conditional models, there were a few significant associations between the covariates and the classes. The findings indicated that compared to the low classes, the high class included more severe TBI and spent fewer days in hospital, and the delayed class also spent fewer days in hospital. Another finding worthy of note is the lack of an association between the classes and severity of TBI, which is consistent with the results obtained by Rapoport (2010) who noted that severe TBI was associated with an increased risk of depression (although not a ‘dose-related’ relationship). Similar findings were reported by Peleg et al. (2009) and Seel et al. (2010). Taken together these findings indicate that spending more time in hospital following TBI can reduce depression for individuals. In addition, the severity of TBI is likely to increase depression levels in those with high levels of depression just after injury.

The current study also examined how the covariates predicted the growth factors within the classes. For the intercepts, the findings for the high class indicated that it was associated positively with age and PTA, and negatively with sex. The investigation of age in adult TBI studies has been limited, and usually has included only narrow age range. For example, in the study by Rapoport et al. (2005), their TBI group had a mean age of 34.9 years, and all participants were younger than 65 years. For the delayed class, the intercept was associated negatively with age and PTA, and for the low class it was associated negatively with sex and positively with PTA and age. Taken together these findings indicated that for the high class, higher initial level of depression was associated with being older, female, and having a severe TBI. For the delayed class, higher initial levels were associated with being younger and having a less severe TBI. For the low class, they were associated with being female and having a more severe TBI.

For the high class, the linear parameter was associated positively with age, sex and duration of hospital stay, and the quadratic parameter was associated negatively with sex. Thus the slight overall linear decrement in depression over the 24 months noted for this class was associated with being older, male, and severe TBI; the slight non-linear increase in depression over the 24 was associated with being female. For the delayed class, the linear parameter was associated positively with PTA and negatively with number of days in hospital. Thus, the overall linear increase in depression in this class was associated with severe TBI and fewer days in hospital. The quadratic parameter had no association with any covariate. The linear parameter for the low class was associated positively with sex, and the quadratic parameter was associated negatively with sex: the overall linear decrement in depression, and the slight non-linear increase in depression over the 24 months in this class were associated with being female.

We also examined the differential association of the classes with the different causes of TBI. The findings showed that the low class had more individuals from fall and sport injuries than that expected by chance. The high class had more individuals from assault than that expected by chance, and the delayed class had more individuals from motor vehicle accident than that expected by chance. Thus, there is higher likelihood that individuals who experience TBI from fall and sport will experience low depression post-TBI. There is also a higher probability that individuals who experience TBI from assault will experience high depression post-TBI. Finally, there is a higher likelihood that individuals who experience TBI from motor vehicle accident will experience delayed depression post-TBI.

Although no previous study has used GMM to examine the trajectory classes of depression following TBI, at least two other studies have examined this for other forms of trauma (deRoon-Cassini et al., 2010; Bonanno et al., 2012). Bonanno et al. (2012) examined the trajectory of depression symptoms, derived from the HADS over 24 months following spinal cord injury. Given that the current study examined depression trajectory classes based also on the HADS and also over a period of 24 months, it is interesting to compare the classes obtained here with the classes reported by Bonanno et al. (2012). Unlike the findings in the current study, results in the Bonanno et al. (2012) study revealed four (and not three) classes. Like the current study, they found high, delayed, and low classes. Unlike the current study, they also found a fourth class, with high initial level of depression that decreased to low level by the 24th month (recovered class). Another difference worthy of mention is the shape of the delayed classes. In the current study, the trajectory increased continuously from just after injury to 12 months and then decreased to around their initial levels at 24 months, with the trajectory staying around or above the clinically relevant cut-off score for most of the 24 months. In contrast, in the Bonanno et al. (2012) study, initial depression scores for the delayed class were notably below the clinical level, and while they increased over the first 12 months, there remained at about the 12-month level for the remaining 12 months. Taken together the findings in the current and the Bonanno et al. (2012) studies raise the possibility that while most individuals across different types of trauma would experience continuously low levels of depression following the trauma, trajectory classes for depression following trauma is likely to be trauma specific.

Related to the findings in the current study it is worth that Bombardier et al. (2016), used latent class growth curve analysis to examine depression trajectories over a 1 year interval following mild to severe TBI. Both the unconditional and conditional models resulted in four trajectory groups: low depression, delayed depression, depression recovery, and persistent depression, with low depression (like in the current study) being the largest class. Compared to the low depression group, the other three groups were more likely to have pre-injury history of mental health disorders, drug and alcohol abuse, and on Medicare rather than commercial insurance. Also of relevance, Juengst et al. (2015) used LCGA to examine life satisfaction trajectories over a 5 year interval (three time points) following moderate to severe TBI. Both the unconditional and conditional models resulted in four trajectory groups: stable satisfaction, stable dissatisfaction, initial dissatisfaction improving, and initial satisfaction declining, with stable satisfaction being the largest group. Age, depressive symptoms, cognitive disability, and life role participation as a worker, leisure participant, and/ or religious participant at 1 year post-injury significantly predicted trajectory group membership.

### Clinical Implications

The findings in the current study have clinical implications. They show considerable fluctuation of depression over time, suggesting that clinicians should monitor depression continuous over an extended period (probably for 2 years), and that a non-clinical level of depression at any one point in time does not necessarily mean that the person will continue to have non-clinical levels in the future.

Like the current study, the two previous GMM studies that examine the trajectory classes for other types of trauma also found that the largest class is the low class (deRoon-Cassini et al., 2010; Bonanno et al., 2012). According to these researchers the low class represents a resilient group, suggesting that most TBI survivors (approximately 75%) would not experience clinical levels of depression following their injuries. The findings in the current study indicated that these are more likely to be individuals who show low levels of depression just after injury, have a less severe TBI, spend longer periods in hospital, and have injuries from falling or from sport. In contrast to this group, the remaining 25% may need psychological support as part of their rehabilitation. These are most likely to be individuals with clinically relevant levels of depression just after injury, have more severe TBI, spent fewer days in hospital, and with injuries from assaults and MVA.

### Strengths and Limitations

In summary, this study revealed low, high and delayed class for depression over 24 months following TBI. It also showed that these classes were shaped by number of days in hospital, PTA, and the cause of injury. The different classes and their trajectories found can help to explain contradictory findings for depression noted in previous studies (Hibbard et al., 1998; Deb et al., 1999; Kreutzer et al., 2001; Fleminger et al., 2003; Ashman et al., 2004; Jorge et al., 2005; Whelan-Goodinson et al., 2009; Koponen et al., 2011). It is important to note that we were able to identify the classes and bring clarity to the existing literature because we applied GMM. This would not have been possible if we treated individuals with TBI as a single group, and used mean level data or percentages above clinical cut-off scores, as adopted in previous studies.

Although the findings here provide new and valuable data on TBI, there are a number of limitations to consider. First, as our scores for depression were based on self-ratings, the ratings may be confounded by common method biases. Second, without pre-injury scores for depression, the extent to which the trajectories found were influenced by participants’ pre-injury levels of depression is unknown. Third, the outcomes of TBI are related to the combined effects of pre-morbid, injury-related, and post-injury factors. Among the pre-morbid factors not considered in the current study are personality, social role, social support, education, IQ, psychiatric and substance abuse problems prior to injury, psychological coping liabilities, length of coma, litigation and disability (Levin et al., 1982; Thomsen and Jensen, 1991; Kay et al., 1992; Ruff et al., 1993; Van Reekum et al., 1995; MacMillan et al., 2002; Demakis et al., 2010; Rao et al., 2010). As only a limited number of covariates were studied, we do not know how the exclusion of the other covariates influenced the trajectory classes. Fourth, as the impact of TBI on depression may be greater in older age, the use of a sample with a wide age range could be seen as problematic. However, this is unlikely as we controlled for age in the unconditional GMM, and there was no significant association between age and class. Fifth, although the final model was based on fit statistics and empirical grounds, we did not do so with covariates also included, as has been suggested by Muthén (2004). Like previous GMM studies involving other forms of trauma (deRoon-Cassini et al., 2010; Bonanno et al., 2012), we did not include covariates when determining classes because our use of covariates was primarily exploratory and not guided by strong theory. Given these limitations, it is proposed that more GMM studies be conducted in this area controlling for these limitations.

## Author Contributions

RG lead role in statistical analysis, designing this specific study, and drafting the paper. CS major role in designing the overall project, data collection, and drafting the paper. MT major role in data collection, minor role in drafting the paper. MS major role in designing the overall project, minor role in drafting the paper.

## Conflict of Interest Statement

The authors declare that the research was conducted in the absence of any commercial or financial relationships that could be construed as a potential conflict of interest.

## Footnotes

1 We also examined anxiety. All findings were highly similar to that reported here for depression. In view of this and because of space limitations, details are not reported in this paper.
